# Anlotinib for patients with small cell lung cancer and baseline liver metastases: A post hoc analysis of the ALTER 1202 trial

**DOI:** 10.1002/cam4.4507

**Published:** 2021-12-23

**Authors:** Ying Cheng, Qiming Wang, Kai Li, Jianhua Shi, Lin Wu, Baohui Han, Gongyan Chen, Jianxing He, Jie Wang, Haifeng Qin, Xiaoling Li

**Affiliations:** ^1^ Department of Thoracic Medical Oncology Jilin Cancer Hospital Changchun China; ^2^ Department of Internal Medicine Affiliated Cancer Hospital of Zhengzhou University Henan Cancer Hospital Zhengzhou China; ^3^ Department of Pulmonary Oncology Tianjin Medical University Cancer Institute and Hospital Tianjin China; ^4^ Department of Medical Oncology Shandong Linyi Tumor Hospital Linyi China; ^5^ Department of Thoracic Medical Oncology The Affiliated Cancer Hospital of Xiangya School of Medicine Central South University (Hunan Cancer Hospital) Changsha China; ^6^ Department of Respiratory Medicine Shanghai Chest Hospital Shanghai Jiaotong University Shanghai China; ^7^ Department of Respiratory Medicine Harbin Medical University Cancer Hospital Harbin China; ^8^ Department of Thoracic Surgery The First Affiliated Hospital of Guangzhou Medical University Guangzhou China; ^9^ Department of Thoracic Medical Oncology Cancer Hospital Chinese Academy of Medical Sciences Beijing China; ^10^ Department of Pulmonary Oncology The Fifth Medical Centre of Chinese PLA General Hospital Beijing China; ^11^ Department of Medical Oncology Liaoning Cancer Hospital Shenyang China

**Keywords:** adverse events, anlotinib, liver metastasis, small cell lung cancer, survival

## Abstract

Liver metastasis is common in advanced small cell lung cancer (SCLC). There is no evidence‐proven treatment beyond the second line in patients with SCLC and liver metastasis. This study aimed to investigate survival in patients with SCLC and liver metastasis treated with anlotinib compared with placebo. This study was a post hoc analysis of the phase II ALTER 1202 trial, including patients who had liver metastasis at baseline. The participants were randomized 2:1 to receive either 12 mg/day anlotinib (anlotinib group) or placebo (placebo group). Tumor response, progression‐free survival (PFS), and overall survival (OS) were compared. In the original trial, there were 39 participants with liver metastasis at baseline, including 27 and 12 in the anlotinib and placebo groups, respectively. The objective response rate was 3.7% and 0% in the anlotinib and placebo groups, respectively (*p* = 0.9999). An elevated disease control rate was found in the anlotinib group (44.4%) compared with the placebo group (8.3%, *p* = 0.0173). The median PFS was 1.51 vs. 0.71 months in favor of anlotinib (hazard ratio [HR] = 0.365, 95% confidence interval [CI]: 0.17–0.78; *p* = 0.0064), with no marked difference in median OS (3.29 vs. 1.91 months; HR = 0.51, 95% CI: 0.22–1.16; *p* = 0.0996). The most common AEs in the anlotinib group were hypertension (40.7%), fatigue (29.6%), loss of appetite (22.2%), and weight loss (22.2%). There were no grade 5 AE. In conclusion, anlotinib increased PFS compared with placebo in patients with SCLC and liver metastasis.

## Graphical abstract

This is a post‐hoc analysis of ALTER 1202 trial to investigate clinic benefit in patients with SCLC and liver metastases treated with anlotinib compared with placebo. Patients with liver metastases at baseline (27 in the anlotinib group and 12 in the placebo group) are included and significant improved median PFS is shown in anlotinib group.

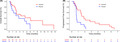

## 1 INTRODUCTION

Approximately 2,093,876 new cases of lung cancer were diagnosed in 2018 worldwide, leading to more than 1,761,007 deaths.^1^ Small cell lung cancer (SCLC) accounts for about 13%–15% of all lung cancers.^2^, ^3^ SCLC is a malignant epithelial tumor consisting of small cells with scant cytoplasm, ill‐defined cellular borders, finely granular nuclear chromatin, and absent or inconspicuous nucleoli. It is characterized by rapid doubling time, high growth fraction, andwidespread metastases early in the disease course, and the patients often present with hematogenous metastases.^4^ Smoking is the main risk factor for SCLC, and current findings indicate that smokers are increasingly prevalent among Chinese patients with SCLC.^5^ According to GLOBOCAN 2018, lung cancer incidence in men is 47.2 per 100,000 in China versus 21.9 per 100,000 in women.^1^ Lung cancer represents 21.9% of all cancers in Chinese men and 13.3% in Chinese women.^6^ Therefore, there are many patients with SCLC in China, accounting for 18.3% of all lung cancer cases in 2010.^7^ SCLC has a poor prognosis, with about 70% of the patients being metastatic at presentation.^8^, ^9^, ^10^ In addition, the median overall survival (OS) is about 8–11 months, and the 5‐year OS rate is <5%.^8^, ^9^, ^10^

Liver metastasis commonly occurs in patients with SCLC and leads to worse OS than patients without liver metastases.^11^, ^12^, ^13^, ^14^ Individuals with advanced and/or metastatic cancer only receive palliative care, usually with chemotherapeutics.^4^, ^13^, ^14^ For patients with SCLC and liver metastasis, there is no evidence‐based drug for third‐ and subsequent lines of treatment.

Anlotinib is an oral tyrosine kinase inhibitor (TKI) that targets the vascular endothelial growth factor receptor (VEGFR), platelet‐derived growth factor receptor (PDGFR), fibroblast growth factor receptor (FGFR), and c‐Kit. It inhibits both tumor angiogenesis and tumor growth and is an approved treatment for advanced NSCLC by the National Medical Products Administration (NMPA), based on the results of the ALTER 0303 study.^15^, ^16^ Anlotinib is currently undergoing careful exploration as a treatment option for SCLC, soft tissue sarcoma, colorectal cancer, and other tumor types. The results of the phase II ALTER 1202 trial of anlotinib for third‐line or beyond treatment in SCLC have been published recently.^17^, ^18^ In that placebo‐controlled, multicenter study, anlotinib markedly improved progression‐free survival (PFS) and overall survival (OS) in patients with SCLC. Nevertheless, investigations are still needed to determine the exact benefits of anlotinib in SCLC.

Therefore, the present study aimed to investigate the survival of patients with SCLC and liver metastasis at baseline after treatment with anlotinib or placebo.

## 2 MATERIALS AND METHODS

### 2.1 Study design and patients

This study performed a post hoc analysis of the ALTER 1202 (NCT03059797), including only the patients with liver metastasis at baseline. The ALTER 1202 trial was a randomized, double‐blinded, placebo‐controlled, multicenter phase 2 trial that compared the efficacy and safety of anlotinib vs. placebo in limited or extensive‐stage SCLC. Originally, the participants underwent randomization into the anlotinib and placebo groups (at a ratio of 2:1), stratified according to the clinical stage (limited vs. extensive) and pattern of relapse after chemotherapy (sensitive vs. refractory relapse). Permuted block randomization with predefined blocks of six was used within each stratification. The randomization process was done centrally using an interactive web response system programmed and managed by a biostatistician in the Department of Biostatistics, School of Public Health Nanjing Medical University. Hence, the patients were assigned to receive anlotinib (recommended dosage of 12 mg p.o. once/day for 14 days every 21‐day cycle) or a placebo. When toxicity occurred, dose reductions to 10 or 8 mg once daily were allowed. The patients were treated until disease progression, intolerable toxicity, or discontinuation at the physician's or patient's request. Crossover was not allowed. The trial had the approval from the ethics committee of each participating center; signed informed consent was obtained from each patient.

The inclusion criteria of the original trial were (1) age of 18–75 years old, (2) histologically confirmed SCLC, (3) progression after ≥2 lines of chemotherapy, (4) Eastern Cooperative Oncology Group (ECOG) performance status score of 0 to 2, (5) a life expectancy >3 months, (6) having ≥1 measurable target lesion as defined by the Response Evaluation Criteria In Solid Tumors version 1.1 (RECIST 1.1), and (7) no organ dysfunction within one week prior to enrollment. In the presence of brain metastases and/or spinal cord compression, the patients were eligible only if they had no symptoms or stable disease under adequate treatments. Liver metastases were confirmed by imaging at baseline.

### 2.2 Endpoints and assessments

The primary endpoint was PFS, defined as the time from the date of randomization to the date of either disease progression (defined by the RECIST 1.1) or death from any cause, whichever occurred first. The secondary endpoints included OS, defined as the time from the date of randomization to the death of death from any causes, objective response rate (ORR), disease control rate (DCR), and safety.

Tumors were evaluated according to RECIST 1.1 by the investigators. Chest, abdominal, and pelvic computed tomography (CT) and/or magnetic resonance imaging (MRI) data were included in the baseline evaluation and subsequent follow‐ups. The efficacy was evaluated preliminary after 3 weeks of treatment and confirmed after 6 weeks, then every 4 weeks (two cycles) until disease progression was confirmed. The adverse events (AEs) and hematological and biochemical toxicities were graded according to the National Cancer Institute Common Terminology Criteria for Adverse Events (version 4.03).

### 2.3 Statistical analysis

In the original trial, the sample size was estimated, assuming a median PFS of 2 and 4 months in the placebo and anlotinib groups, respectively. The original trial aimed to recruit 120 patients (about 90 PFS events).

SAS 9.4 was used for data analysis. All data were from the intent‐to‐treat (ITT) set. Normally distributed continuous variables (Kolmogorov‐Smirnov test) were presented as mean ± standard deviation; those with skewed distribution were presented as median (range). Categorical variables were presented as numbers and percentages. The log‐rank test was used for comparing PFS and OS between the two groups. ORR and DCR were analyzed using the chi‐square test or Fisher's exact test. Hazard ratios (HRs) and 95% confidence intervals (CIs) for PFS and OS were estimated using the Cox proportional hazard model. Variables with *p* < 0.20 in the univariable analyses were included in the multivariable analyses for PFS and OS. All tests were two‐sided (except the chi‐square test), and *p*‐values < 0.05 were considered statistically significant.

## 3 RESULTS

### 3.1 Patient characteristics

There were 39 participants with liver metastasis at baseline in the ALTER 1202 trial. They were assigned to receive either anlotinib (*n* = 27) or a placebo (*n* = 12). The baseline characteristics of the randomized patients are presented in Table 1. The patients in the anlotinib and placebo groups were similar in mean age (57 and 59 years), male proportion (70.4% and 75.0%), and smoking history (63.0% and 66.7%). All cases were stage IV. In the anlotinib and placebo groups, 85.2% and 66.7% were ECOG 1, and 74.1% and 75.0% had two lines of previous chemotherapy, respectively; 63.0% and 33.3% were sensitive to first‐line chemotherapy, and 74.1% and 83.3% had a history of radiotherapy, respectively. The pattern of relapse was refractory in 37.0% and 66.7% of individuals administered anlotinib and the placebo, respectively.

**TABLE 1:** Baseline characteristics of the patients with liver metastasis

Variable	Anlotinib (*N* = 27)		Placebo (*N* = 12)	
	*N*	%	*N*	%
Age (median, years)	57		59	
Sex				
Male	19	70.4	9	75.0
Female	8	29.6	3	25.0
ECOG performance status score				
0	1	3.7	0	0
1	23	85.2	8	66.7
2	3	11.1	4	33.3
Smoking history				
Never	10	37.0	4	33.3
Former	14	51.9	8	66.7
Current	3	11.1	0	0
Previous lines of chemotherapy				
2	20	74.1	9	75.0
≥3	7	25.9	3	25.0
Pattern of relapse from chemotherapy^a^				
Sensitive	17	63.0	4	33.3
Refractory/resistant	10	37.0	8	66.7
Previous radiotherapy	20	74.1	10	83.3

Abbreviations: CR, complete response; ECOG, Eastern Cooperative Oncology Group; PR, partial response.

^a^ Sensitive and refractory/resistant cases were reflected by relapse >3 and ≤3 months, respectively, following first‐line treatment.

### 3.2 Tumor response

Table 2 presents the tumor response data. In the anlotinib group, 0, 1, and 11 patients achieved CR, PR, and SD, respectively; those numbers were 0, 0, and 1 in the placebo group, respectively. The ORR was 3.7% and 0% in the anlotinib and placebo groups, respectively (*p* = 0.9999). Eleven (40.7%) and one (8.3%) participant in the anlotinib and placebo groups had stable disease (SD), respectively. The DCR was significantly higher in the anlotinib group (44.4%) than in the placebo group (8.3%, *p* = 0.0173).

**TABLE 2:** ORR and DCR of the patients with liver metastasis

Assessment	Anlotinib (*N* = 27)	Placebo (*N* = 12)	*p*
PR	1 (3.7)	0	
SD	11 (40.7)	1 (8.3)	
PD	12 (44.4)	7 (58.3)	
NE	3 (11.1)	4 (33.3)	
ORR (CR+PR) (%)	1 (3.7)	0	0.9999^a^
95% CI	(0.09, 18.97)	—	
DCR (CR+PR+SD)	12 (44.4)	1 (8.3)	0.0173
95% CI	(25.48, 64.67)	(0.21, 38.48)	

Abbreviations: CI, confidence interval; CR, complete response; DCR, disease control rate; NE, non‐evaluable; ORR, objective response rate; PD, progressive disease; PR, partial response.

^a^ From the Fisher's exact probability test.

### 3.3 Efficacy

**FIGURE 1:**
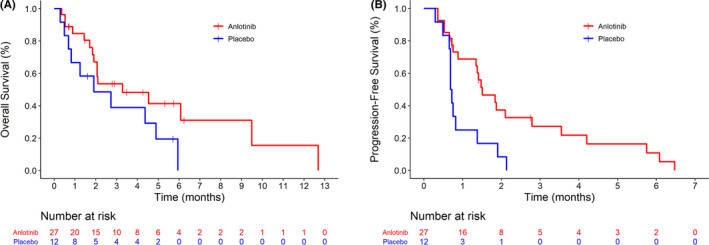
Survival analysis. (A) Overall survival (OS). (B) Progression‐free survival (PFS). Blue solid line, anlotinib group; red dashed line, placebo group

The primary endpoint of this analysis was PFS, with 27 cases assessed in the anlotinib group and 12 in the placebo group. Although mPFS was numerically greater in the anlotinib group (1.51 vs. 0.71 months; HR = 0.365, 95% CI: 0.17–0.78; *p* = 0.0064), this study did not meet the prespecified significance level of 0.05 for OS (3.29 vs. 1.91 months; HR = 0.51, 95% CI: 0.22–1.16; *p* = 0.0996; Figure 1). The Cox multivariable analyses showed that PFS was better in the anlotinib group compared with placebo (HR = 0.437, 95% CI: 0.194–0.984, *p* = 0.046) after adjustment for sex, age, ECOG, smoking history, radiotherapy history, and the number of previous treatment lines; consistent with the non‐adjusted analysis, the adjusted analysis showed no difference in OS (HR = 0.661, 95% CI: 0.261–1.675, *p* = 0.383; Table 3).

**TABLE 3:** Multivariable analysis of PFS and OS

Factors	PFS		OS	
	HR (95% CI)	*p*	HR (95% CI)	*p*
Grouping (anlotinib vs. placebo)	0.437 (0.194–0.984)	0.0455	0.661 (0.261–1.675)	0.3830
Sex (male vs. female)	2.175 (0.519–9.111)	0.2876	0.921 (0.105–8.099)	0.9410
Age (≥55 vs. <55)	1.288 (0.606–2.738)	0.5104	1.317 (0.566–3.065)	0.5235
ECOG performance status (2 vs. 0, 1)	1.431 (0.540–3.789)	0.4711	1.415 (0.497–4.028)	0.5156
Smoking history (yes vs. no)	0.875 (0.237–3.236)	0.8418	1.390 (0.167–11.548)	0.7606
Previous radiotherapy (yes vs. no)	1.028 (0.408–2.593)	0.9532	2.386 (0.755–7.541)	0.1387
Previous lines of chemotherapy (≥3 vs. 2)	1.509 (0.618–3.685)	0.3660	0.582 (0.207–1.633)	0.3035

Note

that variables with *p *< 0.20 in the univariable analysis were entered into multivariable analysis shown here.

Abbreviations: CI, confidence interval; ECOG, Eastern Cooperative Oncology Group; HR, hazard ratio; OS, overall survival; PFS, progression‐free survival.

### 3.4 Safety

The adverse events (AEs) are presented in Table 4. The most common AEs in the anlotinib group were hypertension (40.7%), fatigue (29.6%), loss of appetite (22.2%), and weight loss (22.2%). In the placebo group, the most common AEs were ALT elevation (33.3%), AST elevation (33.3%), and fatigue (25.0%). There were no grade 5 AEs.

**TABLE 4:** Adverse events in patients with liver metastases

	Anlotinib (*N* = 27)		Placebo (*N* = 12)	
	Any grade *N* (%)	Grade 3–4 *N* (%)	Any grade *N* (%)	Grade 3–4 *N* (%)
*Any adverse event*	27 (100)	20 (74.1)	11 (91.7)	7 (58.3)
Hypertension	11 (40.7)	5 (18.5)	1 (8.3)	0
Fatigue	8 (29.6)	0	3 (25.0)	0
Loss of appetite	6 (22.2)	0	2 (16.7)	0
Weight loss	6 (22.2)	0	0	0
Stomachache	5 (18.5)	0	1 (8.3)	0
Hypertriglyceridemia	5 (18.5)	1 (3.7)	1 (8.3)	0
Decreased lymphocyte count	5 (18.5)	2 (7.4)	0	0
Elevated alanine aminotransferase	4 (14.8)	1 (3.7)	4 (33.3)	2 (16.7)
Decreased platelet count	4 (14.8)	2 (7.4)	2 (16.7)	1 (8.3)
γ‐Glutamyl transferase elevated	3 (11.1)	2 (7.4)	2 (16.7)	2 (16.7)
Elevated aspartate aminotransferase	3 (11.1)	2 (7.4)	4 (33.3)	0
Elevated blood alkaline phosphatase	1 (3.7)	0	2 (16.7)	0

## 4 DISCUSSION

SCLC is metastatic at diagnosis in 75% of the cases,^19^ with no evidence‐based therapeutic regimen beyond the second line. Therefore, the present work aimed to assess the survival of patients with SCLC and liver metastasis treated with anlotinib vs. placebo. The present post hoc analysis revealed that anlotinib increased PFS compared with placebo in patients with SCLC and baseline liver metastasis.

Compared with non‐small cell lung cancer (NSCLC), patients with SCLC are more prone to liver metastasis.^20^, ^21^, ^22^ In a population‐based study, 24.3% of the SCLC patients had liver metastasis, and their 3‐year cancer‐specific survival was only 1.7%. Tumor metastasis is related to the microenvironment of the target organ,^23^ and the high propensity of SCLC to metastasize to the liver suggests that the microenvironment of the liver may be more suitable for the survival of SCLC cells with neuroendocrine characteristics compared with NSCLC.^24^, ^25^ Therefore, the natural history of liver metastasis from SCLC is probably different from that of NSCLC, and treatments that are effective for NSCLC need to be validated for SCLC.

Individuals with de novo liver metastases show a poorer prognosis than patients without metastasis or without liver metastasis.^4^, ^20^, ^21^, ^22^ Those with liver metastasis have a worse median OS (3.8 vs. 8.7 months) than patients with no liver metastasis.^26^ Liver metastases from lung cancer can cause bile duct obstruction and affect liver function. In addition, chemotherapy itself can also cause liver damage and affect patient tolerance.^27^ Targeted therapy might be a key factor for improving patient prognosis in SCLC with liver metastasis. Immunotherapeutic drugs, including nivolumab and pembrolizumab, have been approved as a third‐line treatment of SCLC, but lung cancer cases with liver metastases showed reduced anticancer effects (1.4–1.8 months of PFS),^28^, ^29^ which might be related to the liver being an immunosuppressive organ. Nevertheless, immunotherapy alone or combined with other treatments was clinically effective in lung cancer with liver metastases.^30^

TKIs are targeted therapies that prevent the progression of lung cancer.^4^ Anlotinib is an anti‐angiogenic TKI that affects the tumor microenvironment and tumor immunity.^31^ Anlotinib might be effective against lung cancer.^15^, ^16^, ^17^, ^18^, ^31^, ^32^, ^33^ In the present study, 32.5% of the patients had baseline liver metastasis in the ALTER 1202 trial, and PFS was significantly longer in the anlotinib group than in the placebo group. A higher DCR indicates that the anti‐tumor effect of anlotinib in patients with liver metastasis might be the reason for the prolongation of PFS. The results of the full analysis set (FAS) in the ALTER 1202 trial^18^ also showed that the anlotinib group had higher DCR and longer PFS, similar to the efficacy mechanism of anlotinib in patients with liver metastases. Nevertheless, compared with the FAS population in the ALTER 1202 trial,^18^ patients with liver metastases had poorer DCR, PFS, and OS, indicating that the impact of liver metastasis on survival cannot be completely overcome by anlotinib. Only a few studies are available on anlotinib and even fewer for SCLC. This study was the first post hoc analysis of a randomized controlled study specifically focused on a subgroup of patients with liver metastasis. Additional studies are warranted to confirm and refine these results. Furthermore, when the ALTER 1202 trial was designed, there was no standard of third‐line therapy recommended for SCLC, and a placebo was selected as control.^18^ Still, with the development and research on targeted therapies, other drugs might prove beneficial in patients with metastatic SCLC, and comparisons among drugs could be warranted.

The anlotinib group in this study had an AE profile comparable to those reported by previous studies,^15^, ^16^, ^17^, ^18^, ^31^ and anlotinib was well‐tolerated. Interestingly, the incidence rates of increased ALT and AST were lower in the anlotinib group than the placebo group, indicating that anlotinib might have a curative effect on liver metastases while reducing liver function damage. Nevertheless, this requires further investigation.

This study had limitations. The number of patients was small, and the original ALTER 1202 trial was not powered for this post hoc analysis. Larger studies are required to determine the exact benefits of anlotinib in patients with SCLC and liver metastases. Future studies could also examine other metastatic sites.

In conclusion, this study suggests that anlotinib is effective and tolerated in patients with SCLC and liver metastasis. At present, third‐line and later treatment options are limited for SCLC, and there are even fewer treatment options for SCLC combined with liver metastasis. Therefore, the present study provides a potential treatment option for these patients.

## CONFLICT OF INTEREST

The authors declare no conflict of interest.

## AUTHOR CONTRIBUTIONS

Ying Cheng designed the study. Ying Cheng, Qiming Wang, Kai Li, Jianhua Shi, Lin Wu, Baohui Han, Gongyan Chen, Jianxing He, Jie Wang, Haifeng Qin, and Xiaoling Li collected and analyzed the data, and wrote the manuscript. All authors approved the submitted version of the manuscript.

## ETHICS STATEMENT

The ethics committee of each participating center approved the trial, and signed informed consent was obtained from each patient.

## CLINICAL TRIAL REGISTRATION

The present study is a post hoc analysis of the ALTER 1202 trial. This trial was registered with ClinicalTrials.gov identifier: NCT03059797.

## Data Availability

The data that support the findings of this study are available from the corresponding author upon reasonable request.
